# Editorial: From “Cosmic Cooking” to Chemistry of the Future: A Collective Dialogue on Chemistry as a Tribute to Prof. Sourav Pal.

**DOI:** 10.3389/fchem.2022.908165

**Published:** 2022-04-29

**Authors:** Soumyajit Roy, Sailaja Krishnamurty, Wolfgang Schoefberger

**Affiliations:** ^1^ Eco-Friendly Applied Materials Laboratory (EFAML), Materials Science Center, Department of Chemical Sciences, Indian Institute of Science Education and Research, Kolkata, India; ^2^ Physical Chemistry Division, CSIR-National Chemical Laboratory, Pune, India; ^3^ Institute of Organic Chemistry, Johannes Kepler University Linz, Linz, Austria

**Keywords:** theoretical chemistry, cosmic cooking, small molecule activation, coupled cluster and density functional calculations, pincers porphyrinoids and oxometalates, dialogue on chemistry

The Research Topic that you hold in hand was an effort to synthesize a dialogue between Computation and Catalysis so that new insights can be created and new paths paved. It is our view that such dialogue would have been a befitting tribute to Prof. Pal, who has explored, encouraged, and enriched dialogues to create new chemical concepts. The response to this effort is what you are exploring now and what we had the pleasure to curate and edit.

The issue now spans a wide range of explorations: from the early Universe’s “cosmic cooking” with insights into chemical reactions from HeH^+^ to H^3+^ in a study led by the group of Dash et al., to a contribution investigating the source of background cosmic microwave radiation by the group led by Kumar et al. In doing so they compared their results with previous theoretical calculations and analyzed the sources for rotationally excited CN to be behind background cosmic microwave radiation of about 3 K from the interstellar media.

From the cosmic cauldron, we now move to chemical crucibles in the laboratory. The contributions therein can be broadly grouped into catalytic activation of small molecules like that of CO_2_, N_2,_ and H_2_O with an eye to harvesting energy. Agarwal et al. reports the data-driven discovery of 2D materials for solar water splitting. In doing so they employed a conditional variational autoencoder and sampled its latent space to generate several new 2D materials that could likely be effective in carrying out water splitting reactions. The Kumar et al. in their work computationally addresses the solvation of Sc ions with that of water splitting. In another effort, Rohj et al. explores the aspect of ferroelectrics in catalyst design. They demonstrate band gap reduction in BaTiO_3_ through heterovalent doping for potential photocatalyst design. Moving from water to CO_2_ activation, Parmar et al. show the effect of structural details like that of aromatic groups in Mn(I)NNN Pincer complexes on CO_2_ activation using density functionals. They demonstrate that placing an aromatic group at C2-C3 carbons of the Mn(I) NNN Pincers leads to an enhanced effect on CO_2_ hydrogenation. In another review Dedić et al. gives a very detailed account of the state of the art in the matter of Photo and electrochemical CO_2_ activation with metal porphyrinoids. In the matter of N_2_ activation the Choutipalli et al. demonstrated the synergy of doping with N_2_ fixation at the edges of BN nanomaterials. In the context of NRR the Senthamaraikannan et al. used periodic density functionals and proposed a 2-D type catalytic system that has potential for dinitrogen activation. In another work, Das et al. explore cascade catalysis using SOMs as model catalyst systems to demonstrate first polymerization of aniline and then cascading the product catalyst conjugate as a catalyst for oxidation of nitrite to nitrate and aniline to nitrobenzene. The groups led by Raj et al. further take us to the study of gold catalyzed complementary internal redox process of nitroalkyne with a DFT study. Das and Chattaraj comparative theoretical account of electrides take us further down to deeper theoretical explorations in the context of small molecule activation. In another work Yang and Cederbaum, 2022 explores the interesting electronic structural properties of an endocircular Li@C16 to indicates its potential as a catalyst candidate in the future. Tamukong and Hoffmann in his work explores the low lying electronic states of a Ni-dimer and shows certain states to be energetically high lying and ‘van der Waals like in nature’.

Prof. Pal has contributed significantly to the studies of pristine and doped metal clusters in various oxidative catalytic processes, C-X activation, and small molecule activation, including N_2_ activation chemistry. His works on the response properties to multi-reference coupled cluster (MRCC) theory have led to the development of books and book chapters. In this tribute, we have captured the essence of his contribution to chemistry that can nucleate a dialogue on topics ranging from chemistry in the early Universe to the future of our planet with CO_2_, N_2,_ and water acting as feed-stocks for energy.

We hope you find joy as you go through the pages of this issue, and gain as much pleasure we did while curating it.

